# Alexithymia Is Associated with Tinnitus Severity

**DOI:** 10.3389/fpsyt.2017.00223

**Published:** 2017-11-06

**Authors:** Jan Wielopolski, Tobias Kleinjung, Melanie Koch, Nicole Peter, Martin Meyer, Michael Rufer, Steffi Weidt

**Affiliations:** ^1^Department of Psychiatry and Psychotherapy, University Hospital Zurich, University of Zurich, Zurich, Switzerland; ^2^Department of Otorhinolaryngology, University Hospital Zurich, University of Zurich, Zurich, Switzerland; ^3^Neuroplasticity and Learning in the Healthy Aging Brain, University of Zurich, Zurich, Switzerland; ^4^Department of Psychiatry, Psychotherapy and Psychosomatics, Psychiatric Hospital, University of Zurich, Zurich, Switzerland

**Keywords:** tinnitus, alexithymia, Tinnitus Handicap Inventory, Toronto Alexithymia Scale, depressive symptoms

## Abstract

**Objective:**

Alexithymia is considered to be a personality trait with a tendency to express psychological distress in somatic rather than emotional form and, therefore, may play a vital role in somatization. Although, such a propensity can be found in patients suffering from tinnitus, the relationship between alexithymic characteristics and the subjective experience of tinnitus severity remains yet unclear. Our aim was to evaluate which alexithymic characteristics are linked to the subjective experience of tinnitus symptomatology.

**Methods:**

We evaluated tinnitus severity (Tinnitus Handicap Inventory, THI), alexithymia (20-item Toronto Alexithymia Scale, TAS-20), and depression (Beck Depression Inventory, BDI) in 207 outpatients with tinnitus. Correlation analyses and multiple regression analyses were calculated in order to investigate the relationship between alexithymic characteristics, tinnitus severity, and depression.

**Results:**

Highly significant positive correlations were found between THI total score and TAS-20 total score as well as BDI score. Regarding the TAS-20 subscales, multiple regression analyses showed that only the TAS-20 subscale “difficulty in identifying feelings” (DIF) and the BDI significantly predicted the subjective experience of tinnitus severity. Regarding the THI subscales, only higher scores of the THI subscale “functional” demonstrated an independent moderate association with higher scores for DIF.

**Conclusion:**

We found an independent association between the subjective experience of tinnitus severity and alexithymic characteristics, particularly with regard to limitations in the fields of mental, social, and physical functioning because of tinnitus and the difficulty of identifying feelings facet of alexithymia. These findings are conducive to a better understanding of affect regulation that may be important for the psychological adaptation of patients suffering from tinnitus.

## Introduction

Tinnitus is defined as the auditory perception of sound without any corresponding external sound stimulation and occurs in 10–19% of persons in industrialized societies, of which one in five will require medical attention (1–3). It is not completely understood why some persons adapt to their tinnitus symptoms and why others do not (4, 5), but many authors suggest that psychological factors have a notable influence on the subjective experience of tinnitus (6, 7). Langguth et al. proved the importance of anxiety and depression as indicators of experiencing of tinnitus severity by using the Tinnitus Handicap Inventory (THI) (8, 9). Furthermore, a substantial association has been described between tinnitus severity and depression as well as a positive effect of antidepressive treatment on tinnitus severity (10). Moreover, Hiller et al. demonstrated that tinnitus occurred more often in patients with somatization or hypochondriacal disorder and stated that tinnitus may be a somatoform symptom with a possible comorbidity of these different conditions (11). Numerous studies support these suggestions by illustrating similar patterns of subjective loudness and of pitch of tinnitus in patients with great annoyance and in those without annoyance of tinnitus (12–14). A further well-described aspect is the association between tinnitus and reduced quality of life assessed by a standard test procedure (15–17) as well as the association between the greater emotional distress due to tinnitus and the attention that is paid to tinnitus (14).

One condition that may complicate the adaption to emotional distress and lead to a maladaptive coping behavior is alexithymia, which was introduced by Nemiah and Sifneos about 40 years ago based on the clinical observations on patients with psychosomatic disorders (18, 19). Alexithymia is a multifacet personality trait characterized by a reduced ability in identifying and describing one’s feelings, a reduced ability in distinguishing own feelings from bodily sensations, an externally oriented style of thinking, and a restricted imaginal process (20). Alexithymia is associated with increased individual distress (21), reduced health-related quality of life (22), and reduced empathic brain responses (23). Alexithymic people are prone to express psychological distress in somatic rather than emotional form (24), which is considered a triggering factor for psychiatric and behavioral problems such as somatization (24, 25). Congruously, it was found that alexithymia was more prevalent in people with somatoform disorders than in healthy controls (26). These findings are supported by other studies where important factors of alexithymia like difficulties in identifying and describing feelings were related to a greater amount of severe dizziness symptoms (27). Although originally associated with psychosomatic diseases, many studies also already demonstrated a higher prevalence of alexithymia in different psychiatric disorders like panic disorder (28), eating disorders (29, 30), alcohol dependence (31), posttraumatic stress disorders (32), and personality disorders (33, 34) as well as in somatic diseases like inflammatory bowel disease (35), recurrent severe asthma (36), or essential hypertension (37).

Despite numerous publications on alexithymia and somatic symptoms, there are hardly any studies that deal with the associations among alexithymia and tinnitus. As far as we know, only one study exists and has not revealed any correlation between alexithymia and tinnitus severity in a community sample of elderly people aged between 70 and 85 years (38).

However, due to the assumption of the somatoform symptom quality of tinnitus and the mentioned finding that alexithymic characteristics are more prevalent in somatic symptom reporting, our aim was to investigate the relationship between alexithymia and the subjective experience of tinnitus severity in individuals with tinnitus. Furthermore, we wanted to examine which alexithymic characteristics are linked to the subjective experience of tinnitus symptomatology, because they might play an important role for the psychological adaptation of patients suffering from tinnitus.

## Materials and Methods

### Participants

The study was authorized by the ethics committee of the canton of Zurich. Two hundred eighty patients referred to the tinnitus outpatient service at University Hospital Zurich and seen between December 2012 and May 2014 were asked to participate in the study (16). The patients’ medical histories were assessed prior to data recording and all subjects suffering from acute or chronic somatic diseases that could be causing the symptomatology were excluded as well as subjects with chronic psychiatric diseases. All participants gave their written electronic consent before starting to answer the questionnaires online. In case of participants’ questions or uncertainties, a trained medical student provided help in completing the questionnaires. The final sample comprised 207 patients who filled out the questionnaires completely, spoke fluent German, and reported to have had tinnitus for at least 1 month in order to exclude people with temporary symptoms and focus on people with post-acute and chronic tinnitus.

### Measures

To evaluate tinnitus severity, the validated German version of the THI was used, which represents the most standardized tinnitus handicap measuring tool in the literature with excellent internal consistency (Cronbach’s alpha = 0.93) (39, 40). The THI is a self-reported measure consisting of 25 questions grouped into three subscales: functional (11 questions measuring the functional aspects of tinnitus such as mental, social, and physical functioning), emotional (9 questions reflecting affective responses to tinnitus), and catastrophic (5 questions representing catastrophic responses to tinnitus, which include depression and sleep disturbance) (41, 42). Every of the 25 items can be scored with 0 (“no”), 2 (“sometimes”), or 4 (“yes”) points. The total score can be calculated in a range from 0 to 100 and can be subdivided into different grades of subjective experience of tinnitus severity: light (0–16), mild (18–36), moderate (38–56), severe (58–76), and catastrophic handicap (78–100) (43). Furthermore, scores can be calculated for the three subscales: functional (maximum score = 44), emotional (maximum score = 36), and catastrophic (maximum score = 20) (44).

In order to assess alexithymia, the 20-item Toronto Alexithymia Scale (TAS-20) (German version) was administered to the participants (45, 46). The TAS-20, the most commonly used measure of alexithymia, is a valid and reliable 20-item self-report questionnaire with a total score from 0 to 100 and consists of three subscales, measuring the difficulty in identifying feelings (DIF), the difficulty in describing feelings (DDF), and the externally oriented thinking (EOT) (45, 47). There is evidence that the TAS-20 is a reliable and valid measure of alexithymia in normal and clinical adult samples (Cronbach’s alpha = 0.81) (48).

The severity of depression was assessed by the German version of the Beck Depression Inventory (BDI) that consists of 21 items including clinical symptoms of depression with a total score from 0 to 63, with higher scores reflecting higher levels of depression. A total score of 0–10 corresponds with no or minimal depression, of 11–17 with a mild or moderate depression, of 18–63 with a clinical relevant depression. The German version of the questionnaire has shown good psychometric properties (49, 50).

### Statistical Analysis

Descriptive statistics for different measures were calculated for all participants. Data were checked for normal distribution before further statistical analysis. Associations between THI total scores and TAS-20 total scores with subscale scores as well as BDI total scores, and age were tested by using Pearson correlations (two-sided). Afterward, stepwise multiple regression analyses were performed in order to investigate the independent relationship between scores of the TAS-20 with subscales and THI. All statistical calculations were performed using the statistical software package SPSS™/Version 22.0 for Windows (SPSS Inc., Chicago, IL, USA). The significance level was set at *p* ≤ 0.05.

## Results

Seventy-three out of the 207 patients who completed our questionnaires were females (35.3%). Mean age was 46.7 years (SD = 13.9). The average duration of tinnitus was 66.1 months (SD = 92.5). Mean scores on subjective tinnitus severity, alexithymia, and depression (*n* = 207) are presented in Table 1.

**Table 1:** Mean scores on the Tinnitus Handicap Inventory (THI) with subscales, the Toronto Alexithymia Scale (TAS-20) with subscales, and the Beck Depression Inventory (BDI); *n* = 207.

Variable	Mean	SD
THI total	44.4	23.3
THI functional	20.6	11.5
THI emotional	13.4	8.5
THI catastrophic	10.4	4.9
TAS-20 total	44.0	10.8
TAS-20 difficulty in identifying feelings	14.1	5.4
TAS-20 difficulty in describing feelings	11.0	3.5
TAS-20 externally oriented thinking	18.9	4.4
BDI	9.3	6.9

Patients showed, on average, moderate levels of tinnitus severity with a mean THI total score of 44.4 (SD = 23.3). In detail, 26 patients (12.6%) of the sample reported a slight handicap, 60 patients (29.0%) reported a mild handicap, 58 patients (28.0%) reported a moderate handicap, 45 patients (21.7%) reported a severe handicap, and 18 patients (8.7%) reported a catastrophic handicap. These findings are similar to results from other studies (51, 52).

The mean value of the TAS-20 total score in our sample was 44.0 (SD = 10.8), which is slightly higher as compared to the mean score of 39.9 (SD = 8.4) in a representative reference sample of the German population (*n* = 306) (53). Using the TAS-20 cut-off score ≥ 61 (54, 55), 19 patients (9.2%) could be classified as alexithymic, which is consistent with prevalence rates of alexithymia in the German general population (56).

In terms of depression severity, patients showed a mean BDI sum-score of 9.3 (SD = 6.9), which indicates none or minimal depression (57). According to the BDI manual, 133 patients (64.3%) were classified as not depressed, 50 patients (24.2%) were classified as mildly to moderately depressed, and 24 patients (11.6%) were classified as clinically relevant depressed.

Table 2 gives an overview of the Pearson correlations between THI total scores and TAS-20 total scores with subscale scores as well as BDI total scores and age. Highly significant correlations were found between THI total score and BDI score as well as TAS-20 total score and two subscales, measuring the DIF and the DDF (all *p* < 0.01; see Table 2). The third TAS-20 subscale, measuring EOT, and also age did not correlate with THI total score.

**Table 2:** Pearson correlation coefficients between Tinnitus Handicap Inventory (THI), the Toronto Alexithymia Scale (TAS-20) with subscales, the Beck Depression Inventory (BDI), and age; *n* = 207, **p < 0.01.

	TAS-20 total	TAS-20 difficulty in identifying feelings	TAS-20 difficulty in describing feelings	TAS-20 externally oriented thinking	BDI	Age
THI total	0.33**	0.46**	0.28**	0.02	0.70**	−0.09
THI functional	0.33**	0.45**	0.28**	0.04	0.68**	−0.07
THI emotional	0.29**	0.42**	0.26**	−0.02	0.64**	−0.12
THI catastrophic	0.30**	0.41**	0.26**	0.03	0.64**	−0.05

A stepwise multiple regression analysis including all TAS-20 subscales and the BDI score was performed in order to asses for independent relationships between these variables and THI total score (as dependent variable). It was found that only BDI (Beta = 0.64, adjusted *R*^2^ = 0.49, *p* < 0.01) and the DIF-subscale (Beta = 0.12, adjusted *R*^2^ = 0.50, *p* < 0.05) significantly predicted subjective level of tinnitus severity measured by THI.

In order to further estimate the association between tinnitus severity and DIF, a second stepwise multiple regression analysis was calculated with the THI subscales as independent variables and the TAS-20 DIF subscale as dependent variable. The THI total score was not used in conjunction with the THI subscales to exclude redundancy of the data analyses. According to these findings, only higher scores of the THI subscale “functional” demonstrated an independent association with higher scores for difficulty identifying feelings (Beta = 0.45, adjusted *R*^2^ = 0.20, *p* < 0.01).

## Discussion

Our findings establish the existence of a moderate relationship between the subjective experience of tinnitus severity and alexithymic deficits in emotion regulation. More specifically, we found a positive correlation between the functional subscale of the THI, which reflects limitations tinnitus causes in the mental, occupational, social, and physical areas, and the TAS-20 dimension for difficulty identifying feelings. To the best of our knowledge, only one previous study has evaluated the association between tinnitus and alexithymia, but in a community sample of elderly people. In contrast to our findings, Salonen et al. did not find any correlation between TAS-20 scores and tinnitus severity (38). The discrepancy to our results may be explained by the fact that Salonen et al. did not use a standardized instrument to measure tinnitus severity, which was only classified by indicating one of the three groups: no tinnitus, tinnitus without annoyance, and tinnitus with annoyance (38).

Some limitations should be taken into consideration when discussing the results. First, data were collected by self-report questionnaires even though alexithymic patients may have difficulty in adequately assessing their emotional deficits (58). Second, the cross-sectional design of our study precluded any causal interpretation of the relationship between tinnitus severity and alexithymia. We are also quite aware of the fact that the significant association between DIF and THI total score was small in terms of the overall variance explained. Thus, DIF may play a role in the subjective experience of tinnitus severity, but this is not completely confirmed by the actual study. Furthermore, the relation between tinnitus severity and depressive symptoms, which was reported similarly in other studies (43, 59) might be ascribed to a content overlap between the used self-report questionnaires (52). Also further studies are needed to understand the relationship between alexithymic characteristics and the experience of tinnitus severity, which are focused on patients with chronic tinnitus, i.e., tinnitus symptoms for at least 6 months, in order to avoid including patients who are still under posttraumatic distress.

Despite the mentioned limitations our findings suggest that people with difficulties in identifying feelings may tend to experience greater limitations in social, daily, and reading activities involving concentration, auditory acuity, attention, and rest due to tinnitus (as measured by the functional subscale of THI), which is in accordance with De Gucht and Heiser (26). They reported similar outcomes in their review of the empirical literature on somatization and alexithymia: DIF demonstrated the strongest association with the number of symptoms reported, even stronger than the association with general alexithymia. Our results were also consistent with previous investigation that showed that the difficulties identifying feelings factor of the TAS-20 was particularly effective in predicting somatization (60).

Taken together, the disturbances in DIF and the subjective experience of tinnitus severity should be considered in future studies for more precise understanding of this association, preferably with the additional application of observer rated or interview-based methods for measuring alexithymia as the Toronto Structured Interview for Alexithymia (61, 62). Our results benefit a better understanding of emotion regulation difficulties in patients suffering from tinnitus. Furthermore, if replicated, they may have important clinical implications: because people with difficulties in identifying feelings are characterized by using escape-avoidance strategies, individualized psychotherapeutic interventions might potentially benefit these patients. Further research may also point to the fact that not just alexithymic characteristics can predict the experience of tinnitus severity, but a more general impairment in awareness and regulation of mental states (63–65), for example, an impaired self-reflection as found in different psychiatric disorders (66).

## Ethics Statement

The study was authorized by the ethics committee of the canton of Zurich, Switzerland. All participants gave their written (electronic) informed consent in accordance with the Declaration of Helsinki.

## Author Contributions

JW managed data collection, analyzed and interpreted the collected data, and wrote the first draft for the article. TK initiated the collaborative project, conceptualized and designed the project, collected and monitored data collection, and revised the article. MK, NP, and MM collected data, monitored data collection, and interpreted data, and critically revised the draft paper. MR contributed to the concept and design, interpreted data, and critically revised the draft paper. SW initiated the collaborative project, conceptualized and designed the project, designed data collection tools, collected and monitored data collection, interpreted the data, and revised the article. All authors read and approved the final manuscript.

## Conflict of Interest Statement

The authors declare that the research was conducted in the absence of any commercial or financial relationships that could be construed as a potential conflict of interest.
